# Interferon-gamma alters the phagocytic activity of the mouse trophoblast

**DOI:** 10.1186/1477-7827-3-34

**Published:** 2005-08-10

**Authors:** Andréa Albieri, Mara S Hoshida, Sonia M Gagioti, Eduardo C Leanza, Ises Abrahamsohn, Anne Croy, Ali A Ashkar, Estela Bevilacqua

**Affiliations:** 1 Department of Cell and Developmental Biology, Institute of Biomedical Sciences, University of São Paulo 05508-900 São Paulo, Brazil; 2 Department of Immunology, Institute of Biomedical Sciences, University of São Paulo 05508-900 São Paulo, Brazil; 3 Department of Morphology, University of Ibirapuera, São Paulo, Brazil; 4 Department of Obstetrics, Gynecology and Women's Health, University of Medicine and Dentistry of New Jersey, Newark, USA; 5 Depatment of Biomedical Sciences, University of Guelph, Ontario, Canada; 6 Department of Pathology and Molecular Medicine, McMaster University, Hamilton, Ontario, L8N 3Z5 Canada; 7 Present address: Department of Anatomy and Cell Biology, Queen's University, Kingston, Ontario, K7L 3N6 Canada

## Abstract

Interferon-gamma (IFN-gamma) mediates diverse functions in bone marrow-derived phagocytes, including phagocytosis and microbe destruction. This cytokine has also been detected at implantation sites under both physiological and pathological conditions in many different species. At these particular sites, the outermost embryonic cell layer in close contact with the maternal tissues, the trophoblast exhibits intense phagocytic activity. To determine whether IFN-gamma affects phagocytosis of mouse-trophoblast cells, ectoplacental cone-derived trophoblast was cultured and evaluated for erythrophagocytosis. Phagocytic activity was monitored ultrastructurally and expressed as percentage of phagocytic trophoblast in total trophoblast cells. Conditioned medium from concanavalin-A-stimulated spleen cells significantly enhanced trophoblast phagocytosis. This effect was blocked by pre-incubation with an anti-IFN-gamma neutralizing antibody. Introduction of mouse recombinant IFN-gamma (mrIFN-gamma) to cultures did not increase cell death, but augmented the percentage of phagocytic cells in a dose-dependent manner. Ectoplacental cones from mice deficient for IFN-gamma receptor alpha-chain showed a significant decrease of the phagocytosis, even under mrIFN-gamma stimulation, suggesting that IFN-gamma-induced phagocytosis are receptor-mediated. Reverse transcriptase-PCR analyses confirmed the presence of mRNA for IFN-gamma receptor alpha and beta-chains in trophoblast cells and detected a significant increase in the mRNA levels of IFN-gamma receptor beta-chain, mainly, when cultured cells were exposed to IFN-gamma. Immunohistochemistry and Western blot analyses also revealed protein expression of the IFN-gamma receptor alpha-chain. These results suggest that IFN-gamma may participate in the phagocytic activation of the mouse trophoblast, albeit the exact mechanism was not hereby elucidated. Protective and/or nutritional fetal benefit may result from this physiological response. In addition, our data also shed some light on the understanding of trophoblast tolerance to inflammatory/immune cytokines during normal gestation.

## Background

During implantation, mouse trophoblast exhibits an intrinsic potential for phagocytosis, which peaks between days 7 and 9 of pregnancy and is most pronounced in the outermost primary and secondary trophoblast giant cells of the ectoplacental cone [1-4]. This activity declines during a normal gestation, but can resurge under certain conditions [5,6].

Phagocytic activity in post-implantation trophoblast internalizes maternal components, such as uterine epithelial and decidual cells, that are present along the invasion pathway of the trophoblast [3,4]. A role in providing nutrition and space for the early embryonic development is generally attributed to this activity. Intense hemophagocytosis also occurs, which is involved in iron uptake for fetal hemopoiesis [7,8]. Protection against pathogens at the maternofetal interface and immunoregulation of pregnancy has also been implicated as functions for this phagocytosis [6,9-14].

Compared with the phagocytic cells derived from bone marrow, the phagocytic and regulatory processes in the trophoblast have similarities. As with macrophages, phorbol myristate acetate (PMA), all-trans-retinal and complement component 3 enhance trophoblast phagocytosis and trigger the production and release of reactive oxygen species [15-18]. In addition, IFN-γ increases production of nitric oxide by trophoblast cells [19]. The molecular mechanisms involved in the phagocytic process exhibited by trophoblast, however, are not well known. In macrophages, phagocytosis is initiated via a plasma membrane signal that, after activating a JAK-STAT pathway, triggers a sequence of events leading to the internalization of particles [20-22]. Production of IFN-γ by activated, type 1 T lymphocytes and NK cells in response to inflammatory or immune challenges is one of the most effective regulatory signals in this process.

In many different species, IFN-γ is found at the maternofetal interface at specific intervals during normal pregnancy, produced by uterine activated T lymphocytes and natural killer cells [23-26], or even by trophoblast cells [27,28]. In mice, uterine natural killer (uNK) cells seem to be the principal maternal cells producing IFN-γ [24,25,28,29]. The effects of IFN-γ on pregnancy outcomes however, can be pathological or physiological depending upon several factors such as the susceptibility of the mice strain, the concentration of IFN-γ, the stage of pregnancy, the degree of differentiation of the cells at the maternofetal interface and the co-expression with other inflammatory cytokines [30-35]. In vitro, IFN-γ also exhibits a potent ability to induce differentiation in cytokeratin-positive ectoplacental cell populations [36].

Those findings, coupled with the high trophoblast-cell potential for phagocytosis, prompted us to examine a possibility of regulatory roles for IFN-γ in the maternofetal interface. In the present study, we use cultured ectoplacental cone-derived trophoblast to explore the effect of IFN-γ as a phagocytic stimulator at the maternofetal interface.

## Methods

### Mice and collection of ectoplacental cone tissue

To obtain pregnant females, three separate groups of two-month-old mice were mated as follows: a) F1 [NZW × AKR] females with BALB/c males; b) IFN-γRα^-/- ^females with IFN-γRα^-/- ^males; c) 129/SvJ females with 129/SvJ males. The latter two, which are congenic strains, were purchased from the Jackson Laboratory (Bar Harbor, ME, USA) and bred at the University of Guelph, Ontario, Canada. The F1 mice were obtained from Animal Care Facility of the University of São Paulo Institute Of Biomedical Sciences and mated in Sao Paulo. Estrous females were overnight caged with males (1:1) and examined on the following morning for presence of a vaginal plug indicative of mating. The morning of plug appearance was designated gestation day (gd) 0.5. On gd 7.5, mated females were sacrificed by cervical dislocation and their uteri dissected in phosphate-buffered saline (PBS; Gibco BRL, Grand Island, NY, USA) containing bovine serum albumin (BSA). Embryos at the primitive streak stage were then gently extracted from their decidual capsules. Embryonic ectoderm and the extraembryonic membranes (amnion and chorion) were dissected free from the ectoplacental cones and the cones were collected for the experimental protocols. As previously shown through morphological analysis and cytokeratin immunolocalization [18,19], the dissection of the ectoplacental cones at this time of gestation provides pure preparations of trophoblast cells. All procedures were conducted under aseptic conditions using sterile PBS and 10 % fetal bovine serum (wt/vol, FBS).

The Animal Ethical Committees of both the Institute of Biomedical Sciences of the University of São Paulo and the University of Guelph granted approval for the procedures and experiments.

### Ectoplacental cone cultures

Glass coverslips (18 mm in diameter) were placed into the wells of 12-well culture plates, and the wells were filled with 1 mL of standard medium: Dulbecco's Modified Eagle's Medium (D-MEM) (Sigma, Chemical Co., St. Louis, MO, USA), supplemented with 10 % FBS (vol/vol), 0.5 mg/mL lactic acid, 0.2 ng/mL Mito™^+ ^and antibiotics (0.05 mg/mL streptomycin and 50 IU/mL-penicillin). Three to five ectoplacental cones were introduced into each prepared well and cultured under standard conditions (36.5°C, 5 % CO_2 _and humidity) for 48 h to allow the trophoblast cells to attach to the coverslips and begin their outgrowth. Ectoplacental cones from IFN-γR^-/- ^mice and their congenic 129/SvJ controls were only available for experiments involving treatment with mrIFN-γ. Ectoplacental cones of fetuses from the allogeneically mated F1 mice were used in all experiments.

### Baseline and stimulated phagocytic assays

Erythrocytes were used as target cells for trophoblast phagocytosis. The erythrocytes were collected from adult male mice genetically matched to the trophoblast and prepared as described previously [15]. Briefly, the animals were anesthetized and the blood was collected by cardiac puncture. The blood sample was then transferred to heparinized 1.5-mL tubes (Liquemine, Roche Quim., Rio de Janeiro, Brazil), and centrifuged to isolate the erythrocytic fraction. Plasma and white blood cell fractions were discarded and the erythrocytes were harvested and re-suspended in D-MEM. After several cycles of centrifugation and re-suspension in D-MEM, erythrocytes were re-suspended. This was done in order to remove any remaining plasma or white blood cell contamination. The erythrocytes were then counted using a hemocytometer and added to cultures at a concentration of 2 × 10^7 ^cells/mL.

Ectoplacental cones were cultured 48 h prior to addition of either erythrocytes or stimulatory agents. The medium was then replaced with 1 mL of standard medium containing the agents to be evaluated (as phagocytic stimulators) and the erythrocytes. This combination was then cultured for an additional 24-h period. At the end of the culture period, specimens were processed for light and electron microscopy.

Erythrocytes were added to all cultures. For stimulation of erythrocyte uptake by trophoblast cells, three experimental groups were performed: i) Concanavalin A (Con A)-stimulated leukocyte-conditioned medium (LCM); ii) LCM+ antibody against IFN-γ and iii) mouse rIFN-γ (proven lipopolysaccharide and endotoxin free, Sigma Chemical Co., St. Louis, MO, USA). The (i) Con A-stimulated LCM was prepared from 24-h cultures of Balb/c splenocytes treated with 5 μg/mL Con-A and added (at 200 μL/mL) to the ectoplacental cone cultures. The (ii) LCM+ antibody against IFN-γ was produced by incubating 200 μL of LCM with monoclonal anti-IFN-γ (XMG 1.2, 200 μg/mL; ^Ab^IFN-γ) for 15 min at room temperature prior to its addition to the culture. The (iii) mrIFN-γ was used in the cultures at a concentration of 100 U/mL of medium. Non-stimulated cultures, containing only standard medium, were analyzed as controls in each experiment. Control cultures were also carried out using standard medium (without IFN-γ) and either an irrelevant antibody (rat IgG anti-CD44 glycoprotein) or monoclonal anti-IFN-γ (XMG 1.2) at concentrations of 200 μg/mL.

The concentration of mrIFN-γ was established from dose-response curves based on phagocytic indices evaluated at concentrations of 10, 100 and 500 U/mL. The final values for m rIFN-γ were selected on the basis of maximal dependent stimulation for erythrophagocytosis. The neutralizing dose of ^Ab^IFN-γ (200 μg/mL) was estimated based upon dose-response curves (0, 20, 50, 100 and 200 μg/mL) as the dose capable of neutralizing the maximal phagocytic activity in the ectoplacental cone cells.

A minimum of three independent experiments made in triplicate was used for each experimental treatment.

### Light and transmission electron microscopic analysis

After the phagocytic assays, at least two samples from each experimental group were fixed in 2 % paraformaldehyde (wt/vol, EMS, Fort Washington, PA, USA) in 0.1 M PBS, pH 7.2. Fixed cultures were either stained with toluidine blue and observed under light microscopy or processed for cytokeratin A and IFN-γα chain receptor immunohistochemistry localization. In addition, one unfixed sample per group was also stained with YOPRO-1 for apoptosis detection.

For the remaining samples, the ectoplacental cones were first scraped from the culture wells and then fixed in 2.5 % glutaraldehyde (vol/vol, Sigma Chemical Co., St. Louis, MO, USA) in 0.1 M PBS, pH 7.4, for 30 min. The samples were post-fixed in osmium tetroxide (Polysciences, Warrington, PA, USA) in PBS, dehydrated in a graded ethanol series and embedded in Spurr's resin. Sections of 1-μm thickness were cut, stained with toluidine blue and examined under light microscopy. Thin sections were also prepared and stained with 2 % uranyl acetate (wt/vol) and 0.5 % lead acetate (wt/vol) before examination with a Jeol CX-100 transmission electron microscope.

All experiments were prepared and performed in triplicate on three different occasions so that, from each experimental group, 9 samples, containing an average of 27 ectoplacental cones, were available for quantitative analysis.

### Measurement of phagocytic activity and statistical analysis

Ectoplacental cones were completely sectioned in semi-serial 1 μm-thickness sections. From each sample, the slide showing the greatest portion of the ectoplacental cone was selected for quantitative analysis. Phagocytic activity was then measured in toluidine blue-stained sections in a Nikon Optiphot light microscope, employing a 40x objective at a final magnification of × 400. Trophoblast erythrophagocytosis was estimated as the percentage of trophoblast giant cells (TGC) that contained one or more erythrophagosomes.

The phagocytic index (PI) was calculated as follows:



The PIs of ectoplacental cones sharing the same well were analyzed and presented as a group mean (*n*). For each experimental variant, a minimum of 3 groups was analyzed and the results presented as mean ± SD. Differences among the groups were analyzed by ANOVA followed by Tukey-Kramer multiple comparisons test or Student *t*-test. Differences were considered significant at *p *≤ 0.05.

### Immunohistochemistry

Ectoplacental cone cultures grown on glass coverslips were washed in PBS and fixed for 10 min at -20°C in 1 % paraformaldehyde in methanol (vol/vol). After fixation, the cells were washed in Tris-buffered saline (TBS), pH 8.2, at 4°C and then immunostained using the following incubation sequence: a) TBS-0.2 % glycine (vol/vol) for 10 min at room temperature, followed by acetone at -20°C for 15 min and TBS-0.05 % saponin (wt/vol, for recovery of antigenic sites) for 5 min; b) 5 % aqueous acetic acid (wt/vol, to block endogenous alkaline phosphatase activity) for 8 min; c) 1 % goat non-immune serum (wt/vol) at 37°C for 1 h; d) biotin-conjugated monoclonal rat anti-mouse CD 119 (1:10, IFN-γ receptor α chain-GR20; BD Biosciences, Palo Alto, CA, USA) or rat anti-mouse A cytokeratin (1:5, cytokeratin Endo-A; Developmental Studies Hybridoma Bank, Iowa City, IA, USA) at 4°C overnight; e) biotinylated goat anti-rat IgG (1:300, Vector Lab., Burlingame, CA, USA) at 37°C for 1 h. The reaction signal was amplified using the ABC system (Biomeda, Foster City, CA, USA), revealed with fast red/naphthol (Sigma) and counterstained with Mayer's hematoxylin. Substituting normal rat serum for the primary antibodies carried out negative control.

### Western Blot analysis

In two independent experiments, total protein extracted from 121 cultured ectoplacental cones was mixed with an equal volume of SDS-PAGE sample buffer, boiled for 10 min, and then separated on 12 % SDS-PAGE gels. After electrophoresis, proteins were transferred to membranes (Hybond-ECL; Amersham, UK), which were then blocked in 5 % dry milk in TBS-Tween 20 (1 h) at room temperature, rinsed, and incubated (12 h, 4°C) with the primary antibody rat monoclonal IgG anti-IFN-γ receptor α chain (GR20; 2.0μg/mL in TBS; BD Biosciences, Palo Alto, CA, USA). The primary antibodies were removed and the membranes were washed in TBS and sequentially incubated with TBS-Tween 20-anti-rat IgG-biotinylated secondary antibody (1:1000, Vector Lab., Ca, USA), and with avidin conjugated to horseradish peroxidase (Vectastain ABC kit, Vector Lab., Ca, USA) according to manufacturer's instructions. The membranes were washed again and the bound enzyme detected by chemiluminescence following the manufacturer's protocols (Amersham Biosciences, UK).

### Analysis of cell death

Detection of cell death was carried out using the fluorescent probe YOPRO-1 (Molecular Probes, Eugene, OR, USA), which selectively binds to apoptotic nuclei (37). After the stimulatory assay using different concentrations of mrIFN-γ (0, 100, 1,000 and 10,000 U/mL), treated and untreated cultures were washed in standard medium and incubated with YOPRO-1 (10 μM/mL in the same medium, for 10 min). Apoptotic cells were identified by green fluorescent nuclei on a Zeiss confocal laser scanning microscope (Zeiss LSM 510, EXmax/Exmin = 494/518 nm), using the 488-nm line of the Argon laser and connected to an inverted fluorescence microscope (Zeiss Axiovert 100 M). The number of reactive cells per ectoplacental cone in three independent experiments was analyzed and the results presented as mean ± SD. per experimental treatment. Differences among the groups were analyzed by ANOVA followed by Tukey test and considered significant at *p *≤ 0.05.

### Semiquantitative RT-PCR analysis

RNA was isolated from ectoplacental cones cultured with or without 100 U/mL IFN-γ. The cultures were washed using 0.1 M PBS and total RNA was isolated using Trizol (Gibco BRL) according to manufacturer's instructions. The RNA was diluted in diethyl pyrocarbonate-treated (DEPC)-water, quantified by spectrometry and used for reverse transcriptase PCR (RT-PCR).

Reverse transcriptase and PCR assays were performed according to manufacturer instructions, employing the Superscript Preamplification System for First Strand cDNA Synthesis (Gibco BRL). Briefly, cDNA was synthesized from 1 μg of total RNA after priming with oligo (dT). The final reaction volume was diluted to 20 μL, and 2 μL of each cDNA sample were used as a template for gene-specific PCR amplifications. PCR amplification was performed in a 50-μL final reaction mixture using a thermocycler (BioRad, Hercules, CA, USA). Cyclophilin was used as a gene amplification control and as the reference for quantitative analysis. The PCR products were separated by electrophoresis using a 1 % agarose gel and revealed through ethidium bromide staining. The PCR cycle consisted of an initial single denaturation at 94°C for 5 min, followed by denaturation at 94°C for 30 sec, annealing at 60°C for 30 sec and elongation at 72°C for 60 sec. A total of 30 cycles were run. Cycle number was selected to allow quantitative comparison of the samples in a linear way. The primers used for the amplification of IFN-γRα and β chain primers (Life Technologies, Gibco BRL, NY, USA) were those described by Lucas et al. [22] and contained in the Gene Bank database of the National Center for Biotechnology Information . The primers sequences and the respective fragment lengths are: IFN-γRα (608 bp): sense – 5'-CGGTCGAAAAAGAAGAGTGTA-3'; antisense – 5'-tcgggagtgataggcggtgag-3', IFN-γRβ (528 bp): sense- 5'-TACACTTCTCCCCTCCCTTTG-3'; antisense- 5'-ACATCATCTCGGTCCTTTTCT-3'. cyclophilin [[38], 668 bp] sense- 5'-CTTGCTGCAGACATGGTC-3'; antisense- 5'-GCAATCCTGCTAGACTTG-3'.

The density of each band was measured using a Kodak Digital Science™, ID Image Analysis Software (Eastman Kodak Company, Rochester, NY) and expressed as optical density. Data are presented as a ratio compared to cyclophilin expression. This particular approach to semiquantitative RT-PCR was based upon analytical data published by Kinoshita et al. [39]. Statistical analysis of the data for each gene was performed using the Student's *t*-test to compare two groups. Data are presented as mean ± SD. A value of *p *< 0.05 was considered to indicate a statistically significant difference.

## Results

### Trophoblast cell characteristics in IFN-γ-treated and untreated cultures

**Figure 1:**
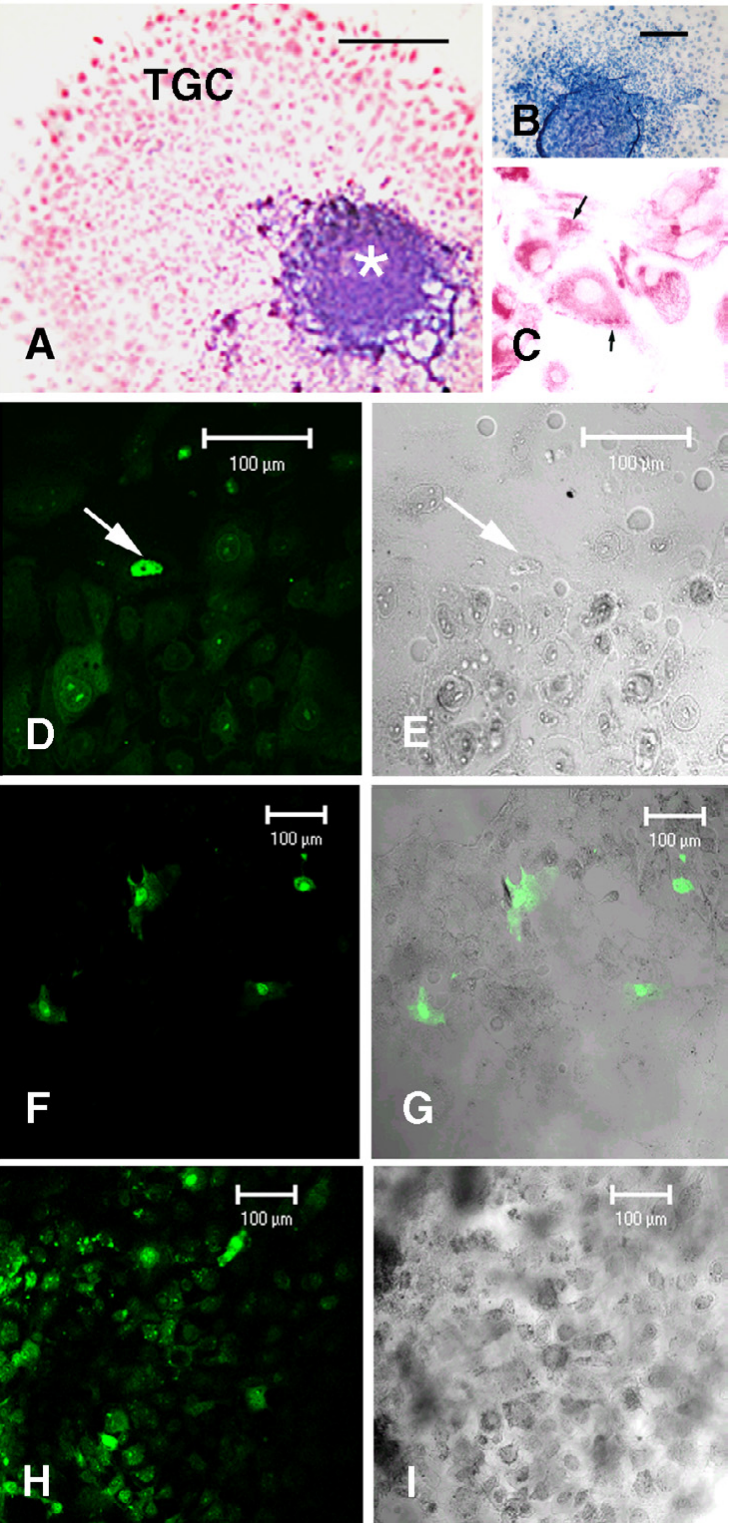
**Forty eight hour-cultured ectoplacental cone cells. **Samples were stained for cytokeratin-A intermediate filaments typical of trophoblast cells (**A-C**) or YO-PRO to localize cell death (**D-I**). IFN-γ treatments were: **D-E**, no treatment;**F-G**, 100 U/mL and,**H-I**, 10,000 U/mL. The arrows in figure **C **highlight cytoplasmic filaments and in figures **D-I**, dead cells. **A-C**, Alkaline phosphatase immunostaining. **B**, Negative control in which the primary antibody was omitted. **D**, **F **and **H**, Fluorescence microscopy. **E**,**G **and **I**, Nomarski imaging of **D**,**F **and **H**, respectively. The unique bar in figure **A **represents: 300 μm in **A**, 160 μm in **B**, and 80 μm in **C**.

The general morphology of cultured ectoplacental cone-trophoblast cells was similar to that described previously [15,18]. After 48 h of culture, the peripheral trophoblast cells had already formed an extensive monolayer of large cells and surrounded a core of small cells. Cytokeratin A was detected in all cells of the ectoplacental cone, indicating that only trophoblast cells had been isolated and cultured (Figs. 1A–C). Fluorescent staining with YOPRO-1, nuclear dye that does not label living cells, showed few trophoblast-giant cells in the process of programmed cell death in the control (1.66 ± 0.57/ectoplacental cone) and IFN-γ-treated groups (Figs. 1D–I) at concentrations of 100 and 1,000 U/mL (respectively, 2.33 ± 0.57 and 3.33 ± 1.52/ectoplacental cone). At concentrations of 10,000 U/mL, the dying cells increased (12.33 ± 2.51/ectoplacental cone).

**Figure 2:**
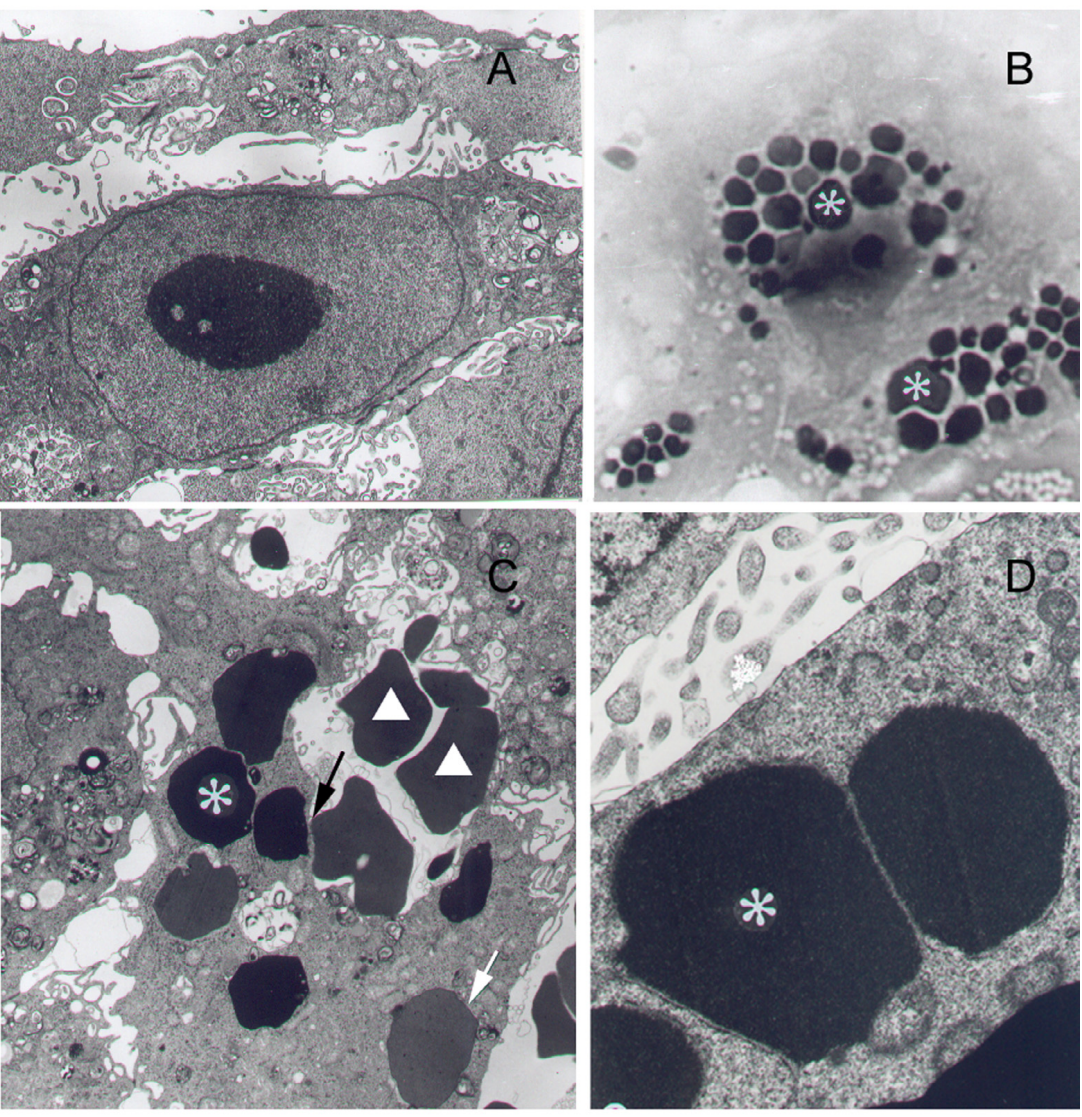
**Cultured trophoblast cells from NZW × AKR F1 (A-C) and IFN-γRα^-/- ^mice (D). **(**A**) Cells of ectoplacental cone cultured in the presence of erythrocytes in standard culture medium show no phagosomes (final magnification, × 8,500), (**B-D**) whereas the cytoplasm of trophoblast cells stimulated with mrIFN-γ exhibit large numbers of erythrophagosomes (*). In the electron micrograph (**C**), different stages of erythrocyte internalization are seen (↑). (▲, free erythrocytes) (**B**, toluidine blue, × 640; **C**, × 5,500; **D**, × 16,500).

Trophoblast cells from either IFN-γRα^-/- ^mice (Fig. 2D) or from normal animals (Figs. 2A–C), from LCM mrIFN-γ treated and untreated groups showed very similar cellular and subcellular characteristics, except for the prominence of different stages of erythrocyte internalisation and erythrophagosomes in the trophoblast giant cells of the treated groups (Figs. 2B–D).

### Trophoblast cell phagocytosis in stimulated cultures

Table 1 provides an analysis of the phagocytic behavior of ectoplacental cones under each treatment employed. The addition of LCM to ectoplacental cone/erythrocyte cultures significantly enhanced erythrophagocytosis in comparison to that observed in non-stimulated control cultures. Use of a neutralizing antibody for IFN-γ significantly reduced LCM-stimulated activity, suggesting that much of the activity in the LCM was due to IFN-γ. In addition, mrIFN-γ also caused a synchronous increment in trophoblast giant cell PI.

**Table 1:** Erythrophagocytic activity of cultured F1-trophoblast giant cells treated with leukocyte conditioned medium and mrIFN-γ

**Culture treatment**	***n***	**PI (%)**
Standard culture medium (SCM)	5	5.1 ± 0.95
SCM + ^Ab^IFN-γ	3	7.14 ± 1.93
SCM + ^Ab^C	4	6.44 ± 3,34
Leukocyte conditioned medium (LCM)	7	38.95 ± 3.50*
LCM+^Ab^IFN-γ	4	18.55 ± 0.22 */**
mrIFN-γ 100 U/mL	3	34.99 ± 1.40*/***

Values significantly different at p < 0.001: *compared to the control containing only standard culture medium and erythrocytes (SCM); ** compared to treatment with leukocyte conditioned medium (LCM) and, *** compared to treatment with LCM (200 μL/mL) plus ^Ab^IFN-γ. PI, phagocytic index calculated as the percentage of trophoblast giant cells containing erythrophagosomes. ^Ab^IFN-γ, monoclonal antibody XMG 1.2 against IFN-γ (200 μg/mL). ^Ab^C, isotype matched control antibody, (200 μg/mL). mrIFN-γ, mouse recombinant IFN-γ (100 U/mL).

**Figure 3:**
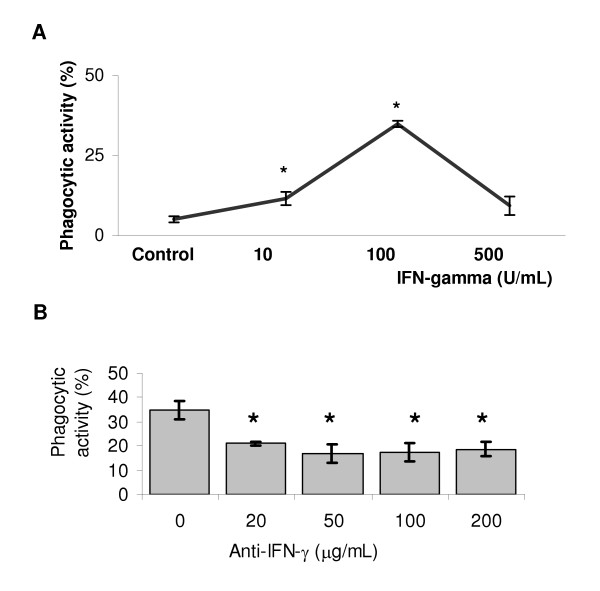
**Effect of (A) mrIFN-γ and (B) monoclonal antibody XMG 1.2 anti-IFN-γ on the erythrophagocytosis exhibited by NZW × AKR F1 gd-7.5 trophoblast cells. **(**A**) Ectoplacental cones were cultured for 48 h in the mrIFN-γ concentrations indicated in the graphics. Note that the highest phagocytic activity occurs at the concentration of 100 U/mL. The cultures that received the monoclonal antibody (**B**, 0–200 μg/mL), also received 200 μL/mL of leukocyte-conditioned medium (LCM). Data represent the mean ± SD of three separate experiments and asterisks show statistical differences in relation to the control (*p *< 0.05).

The doses used for mrIFN-γ were established from dose-response curves (Fig. 3A), which indicated doses of 100 U/mL for IFN-γ as the most effective for enhancing phagocytosis in trophoblast cells. On the other hand, LCM used in combination with different concentrations of the neutralizing antibody against IFN-γ reduced the trophoblast phagocytic response in comparison to LCM alone (Fig. 3B). The predicted effect of immunoglobulin in the phagocytic activity of the trophoblast was also tested using a non-relevant antibody or the neutralizing ^Ab^IFN-γ in a standard culture system in the presence or absence of LCM. The phagocytosis indices, however, did not vary significantly in relation to each respective control. In the system in which LCM was introduced, the phagocytic indices remained as high as those found when no antibody was introduced (Table 1).

Trophoblast giant cells from both IFN-γRα^-/- ^and 129/SvJ mice exhibited very low PIs under non-stimulated conditions. The PIs were slightly higher in the IFN-γRα^-/- ^ectoplacental cone cultures supplemented with mouse rIFN-γ but were still lower than PI values seen in trophoblast cells from congenic controls receiving similar treatment (Table 2).

**Table 2:** Effects of mouse recombinant IFN-γ on erythrophagocytosis exhibited by trophoblast cells from IFN-γRα^-/- ^mice

**Culture treatment**		***n***	**PI (%)**
**Rα^-/- ^mice **	SCM (standard culture medium)	4	14.72 ± 4.05
	SCM + mrIFN-γ (100 U/mL)	5	14.99 ± 10.88
**Congenic animals**	SCM (standard culture medium)	4	11.52 ± 4.65
	SCM + mrIFN-γ (100 U/mL)	3	33.46 ± 5.72 *, **

* Values significantly different compared to cultures using IFN-γ-treated and untreated Rα^-/- ^mice (p <0.05), **compared to cultures of congenic animals that did not receive IFN-γ (p < 0.01) (ectoplacental cone from wild type mice). PI, phagocytic index calculated as the percentage of trophoblast giant cells containing erythrophagosomes. SCM, standard culture medium.

### Analysis of the expression of IFN-γRα and β chains in trophoblast cell cultures

To evaluate whether the phagocytic response of trophoblast was mediated via IFN-γ receptors, we evaluated receptor expression at the mRNA and protein levels.

**Figure 4:**
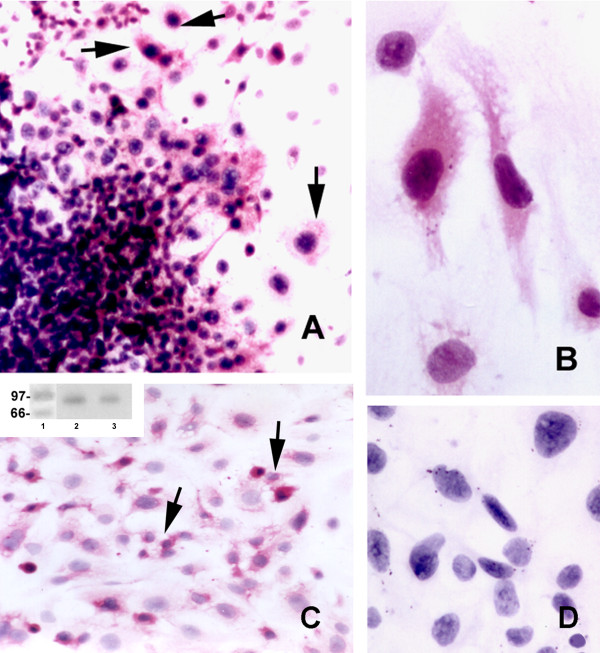
**Protein expression of IFN-γ receptor α chain in cultured ectoplacental cone cells. **Photomicrographs **A, B **and **C **show trophoblast reactive cells (arrows) for IFN-γ receptor α chain in mrIFN-γ-treated (**A-B**) and non-treated cultures (**C)**. (**D**) Control reaction in which, rat non-immune serum was substituted for the primary antibody. No positive cells were observed. The insert in panel C (upper left) displays a Western blot analysis for IFN-γ receptor α chain protein expression. Lane 1 contains the molecular weight marker and Lanes 2 and 3 total proteins from 121-cultured ectoplacental cones probed with anti-IFN-γ receptor α chain antibody (**A**, **C**, × 100; **B**,**D**, × 880).

IFN-γ α-chain receptor was immunolocalized mainly to spread trophoblast cells. Many had giant cell features (Fig. 4). In the proliferative central region of the cultured ectoplacental cone, very few cells were reactive. This pattern of reactivity was maintained in cultures exposed or not exposed to IFN-γ.

**Figure 5:**
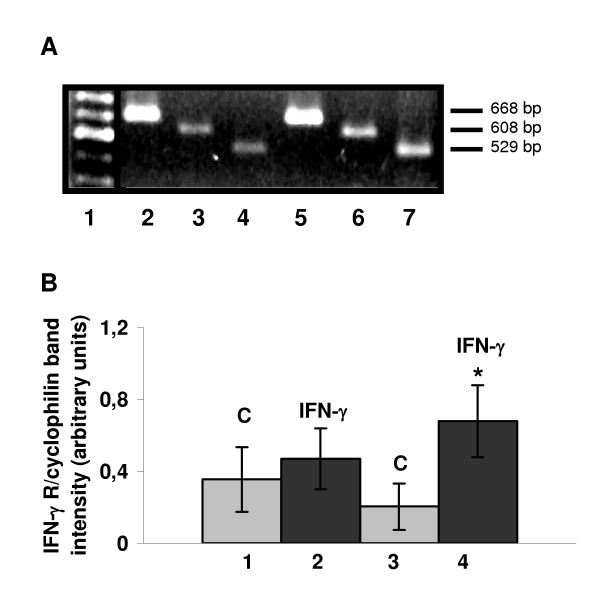
**Gene expression of IFN-γα and β chain receptor mRNA in cultured ectoplacental cones. **(**A**) A representative RT-PCR analysis of mRNA transcript in ectoplacental cone outgrowth cultures lacking (lanes 2 to 4) or with (lanes 5 to 7) mrIFN-γ. Lane 1 represents the DNA ladder, lanes 2 and 5, cyclophilin, used as an internal cDNA control (668 bp), lanes 3 and 6, IFN-γ receptor α chain (608 bp) and lanes 4 and 7, IFN-γ receptor β chain (529 bp). (**B**) Semi-quantitative RT-PCR analysis of IFN-γ receptor chain expression. A summary of 3 experiments is shown in the bar graph (mean and S.D.). Transcripts were expressed as a ratio between band intensities of the IFN-gamma receptor chain and cyclophilin. Asterisk shows statistical differences in relation to the control (c) in which no IFN-γ has been added (p < 0.05). Numbers represent: 1 and 2, IFN-γ α chain receptor expression in the absence (c) and presence of IFN-γ in the culture medium, and 3 and 4, IFN-γ β chain receptor expression in the absence (c) and presence of IFN-γ.

Total RNA was isolated from ectoplacental cones cultured with or without IFN-γ. This RNA was used for RT-PCR evaluation IFN-γRα and β chains. mRNA for both receptor chains (α and β) was detected in trophoblast. In addition, semi-quantitative analyses for both receptor chains normalized against cyclophilin expression demonstrated elevated transcription after mrIFN-γ treatment. Greater expression of IFN-γ receptor β chain than α chain was observed (Fig. 5).

## Discussion

This study confirms data from the literature showing that trophoblast is able to react to immune regulatory molecules by altering its phagocytic behavior [5,11]. Here, we showed that mouse ectoplacental-cone cultures treated with either Con A-activated leukocyte-culture supernatants or low doses of mrIFN-γ contain more trophoblast cells in active phagocytosis than untreated control cultures. Among cytokine mixture present in culture supernatants from stimulated leukocytes, special emphasis was given to IFN-γ and its roles. The phagocytic activity of treated trophoblast cells was specifically neutralized in the presence of antibodies against IFN-γ. This supported the hypothesis that in the conditioned medium, IFN-γ was the molecule most effective in mediating trophoblast erythrophagocytosis. Direct use of low doses of mrIFN-γ in the trophoblast cultures extended this hypothesis.

On the other hand, in the present study we cannot role out the possibility of other unidentified regulatory molecules might be also involved, since we find that the phagocytic activity of ectoplacental cone cells relevantly decreases, but not to control baseline levels in the presence of leukocyte conditioned medium plus neutralizing antibody.

Another physiological question addressed herein was the pathway by which IFN-γ acts on trophoblast cells. Expression of IFN-γ receptor chains was demonstrated at both the mRNA and protein levels suggesting that trophoblast phagocytic activity in the presence of IFN-γ is a receptor-mediated response. A second set of experiments, employing mice that do not express the IFN-γ receptor also predicted indicated the phagocytic response was receptor-dependent. IFN-γR mRNA has previously been detected in pre-implantation mouse embryos and in placentas from gd 14 until birth [40,41]. Taken together with our results, these findings indicate that IFN-γR expression is a fundamental characteristic of trophoblast cells throughout pregnancy. Higher expression of IFN-γ receptor alpha and beta chains in IFN-γ-treated cultures shows that trophoblast cells also respond to IFN-γ in a manner analogous to the responses of macrophages to this cytokine [22].

Although further analyses are required to define the molecular mechanisms involved in IFN-γ-stimulated phagocytosis, our results, suggest that IFN-γ may trigger an autocrine cascade in trophoblast giant cells. IFN regulatory factor-1 (IRF-1) is highly expressed in the mouse trophoblast [42,43]. This IFN-γ-induced transcription factor activates the promoters of many interferon-regulated genes [43], and may a key to understanding the responsiveness of the trophoblast to IFN-γ. Alternatively, IFN-γ stimulated trophoblast cells produce and release nitric oxide [19]. In macrophages, this is one of the most important events triggered by IFN-γ receptor stimulation [44,45].

Despite the numerous studies that show the presence of IFN-γ-producing cells in the pregnant uterus [24,26-29,36,46-48] and, that IFN-γ can be beneficial to pregnancy if secreted at appropriate times, concentrations and locations [29], this cytokine is considered an abortion-inducing factor. [30,32,49-54]. In mice, high, in vivo doses of IFN-γ (3 × 10^5 ^IU/mL) are deleterious to early embryos [55,56] while in vitro treatments reduce trophoblast outgrowth, limiting invasive potential. At low doses (100 IU/mL) IFN-γ increases total cell numbers in cultured ectoplacental cones and promotes their differentiation. In humans, IFN-γ has been associated with direct effects on the viability of cultured term trophoblast cells, whether or not TNF-α was present in the medium [53,57]. Mechanisms participating in IFN-γ-induced fetal death have not been clearly defined and it is quite probable that other types of cells, in addition to trophoblast cells, participate in the in vivo outcomes. Clark et al. found [52,58] that an abnormal increase in endogenous IFN-γ (1,000 IU) combined with high TNF-α (2,000 IU) on gd 7.5 led to abortion within 48 h. These cytokine levels correlated with strong expression of *fgl2 *prothrombinasein decidua as well as in trophoblast suggesting a maternal vascular etiology with thrombosis and ischemia. It is therefore likely that the concentration of IFN-γ, its balance with other pro- and anti-inflammatory cytokines, and the stage of gestation at which is, produced are fundamental in defining whether IFN-γ plays a physiological or pathological role during pregnancy.
